# Osthole Blocks HMGB1 Release From the Nucleus and Confers Protective Effects Against Renal Ischemia-Reperfusion Injury

**DOI:** 10.3389/fphys.2021.735425

**Published:** 2021-12-22

**Authors:** Qing Dai, Deqiong Xie, Chenli Zhang, Lei Zhu, Ying Xu, Kui Li, Wen Hao, Hefei Yin

**Affiliations:** ^1^Department of Nephrology, The Second People’s Hospital of Yibin, Yibin, China; ^2^North Sichuan Medical College, Nanchong, China

**Keywords:** HMGB1, kidney, reperfusion injury, inflammation, histone acetylation, osthole

## Abstract

Renal ischemia-reperfusion (IR) is one of the main causes of renal injury. In severe cases with serious consequences, IR-related renal damage progresses rapidly and can even lead to acute renal failure. Its clinical treatment is currently difficult. According to various studies at home and abroad, HMGB1 is released from the nucleus into the cytoplasm or extracellular space by damaged parenchymal cells during ischemia and hypoxia, and this plays an important role in the initiation of reperfusion injury as an early inflammatory factor and is closely related to the occurrence and development of renal diseases. In recent years, the protective effect of osthole on IR of tissues and organs has been a key topic among clinical researchers. Osthole can inhibit the inflammatory response, reduce cell apoptosis the progression, and improve the prognosis of IR, thus protecting the kidney. During the development of renal IR, finding a mechanism through which the osthole blocks the release of HMGB1 from the nucleus would be helpful in detecting targets for clinical treatment.

## Introduction

The prognosis of ischemia-reperfusion (IR) injury (IRI) is not only closely related to the degree and etiology of renal injury but also depends on the early repair after IR. Our study shows that HMGB1 is an important inflammatory factor in early renal IRI and its release from intracellular to extracellular is affected by various factors. Among them, protein acetylation modification is an important regulatory mechanism, which is very important for the early intervention of HMGB1. Therefore, inhibiting acetylation of HMGB1 and preventing nucleation of HMGB1 may provide a new targeted therapy for alleviating early renal IRI. Osthole, a natural coumarin, is an effective chemical component of hydrocarbon coumarins, with the highest content found in Cnidium monnieri. It has antioxidant, anti-cancer, anti-inflammatory, immunomodulatory, and other effects (Hoult and Payá, 1996; Wang et al., 2007, 2015; Li et al., 2016). In recent years, the protective effect of osthol on tissues and organs after an injury has been the focus of research by scholars at home and abroad. In this study, we explored the mechanism of action of HMGB1 in renal IRI and the protective effect of osthole on IRI to provide a new theoretical basis for the protection of renal IRI through the intervention of osthole on the release of HMGB1.

## Materials and Methods

*Animals*: Sixty male *Kunming* mice, aged 6–8 weeks and without specific pathogens, were obtained from the Laboratory Animal Center of North Sichuan Medical College and were raised in a standard specific pathogen-free laboratory animal feeding environment.

*Reagents*: Osthole with ≥98% purity was purchased from Beijing Solaibo Technology Co. Ltd. (Beijing, China). Creatinine and urea nitrogen assay kits were purchased from Nanjing Jiancheng Institute of Biological Engineering (Nanjing, China). RNA extraction, reverse transcription, and fluorescence quantitative PCR kits were purchased from Vazyme (Nanjing, China). A TRIzol KIT was purchased from Thermo Fisher Inc. (Shanghai, China). HAT, HDAC, NF-KB, TNF-α, IL-6, and HMGB1 enzyme-linked immunosorbent (ELISA) kits were purchased from Elabscience (Wuhan, China). CD4 primary antibodies were purchased from Abcam Company (Shanghai, China). HMGB1, MPO, and CD3 primary antibodies were purchased from Servicebio (Wuhan, China). CD8 primary antibody was purchased from Bioss (Beijing, China). HRP-labeled sheep anti-rabbit secondary antibody was purchased from Wuhan Boxide Biological Engineering Co. Ltd. (Wuhan, China).

Mice in the IRI group were anesthetized by intraperitoneal injection of 100 mg/kg pentobarbital sodium. A longitudinal incision of about 1.5 cm in length was made through the lower margin of the dorsal and bilateral costal arch about 0.5 cm away from the spine, and the posterior peritoneal space was entered. The adipose tissue was bluntly separated, and the left kidney and pedicle were exposed. The left renal artery was isolated and fixed with microascending forceps for 40 min. The left renal artery clip was then loosened to restore blood perfusion to the left kidney. After releasing the artery clip, the kidney changed from purple-black to red, indicating that reperfusion was successful. And the right kidney of the mice was removed at the same time. Finally, the muscular layer and skin of the incision on the back were sutured. At 3, 24, and 72 h after reperfusion, kidney specimens and serum samples were obtained for analysis.

In the sham group, the operation was the same as in the mouse renal IRI group, except that the blood flow of the left kidney was not blocked. Renal tissue and serum samples were collected at 3, 24, and 72 h postoperatively, with six mice at each time point. The mouse renal IRI model was established in the IRI group, and renal tissue samples and serum samples were collected at 3, 24, and 72 h after reperfusion for detection, with six mice at each time point. The optimal postoperative detection time was determined according to pathological injury and biochemical results. In the osthole group, mice received intraperitoneal injection of osthol (5, 10, 20, and 40 mg/kg) 30 min before surgery for renal ISI, with six osthole in each group.

### Blood and Tissue Preparation

At 3, 24, and 72 h after renal blood flow reperfusion, serum and left kidney samples were collected. Part of the left kidney tissue samples was fixed with 4% paraformaldehyde and embedded in paraffin sections. Part of the left kidney and serum were frozen in an −80°C refrigerator.

### Measurements of Serum Creatinine and Blood Urea Nitrogen

The whole blood samples of the mice were placed at room temperature for 2 h, and the supernatant was obtained at 4°C and centrifuged at 3,000 rpm at the separation center for 15 min. The concentrations of serum creatinine (sCr) and blood urea nitrogen (BUN) in serum were determined using an automatic biochemical analyzer. The specific method followed the instruction of the kit.

### Renal Histopathology

The left renal tissue was embedded in paraffin and sectioned with a thickness of 5 μm. Six sections were selected at each time point for observation. Under the optical microscope, 10 non-overlapping fields (200×) were randomly selected from each section for observation and photographs were taken. Sections were stained with hematoxylin and eosin (H&E), periodic acid-Schiff (PAS), and Masson trichrome to evaluate the pathological injury of renal tissue or fiber level under a light microscope. The renal tubulointerstitial semiquantitative scoring method (Men et al., 2016) was used to assign pathological scores to the renal tissues. Inflammatory cell infiltration was scored as follows: ≤25%, 1 point; >25% and ≤49%, 2 points; >49% and ≤75%, 3 points; and >75%, 4 points. The score assigned to mild renal interstitial edema was 1 and that to severe renal interstitial edema was 2. In cases of renal tubular injury, the presence of only epithelial vacuolar granule degeneration was assigned 1 point, the presence of brush edge abscission was assigned 2 points, and the presence of necrosis was assigned 3 points. The semiquantitative score of renal tissue pathological injury was the sum of the above scores. It was scored by the experimenter, intermediate technician, and senior physician of Yibin Second People’s Hospital, and the median value was taken.

### Periodic Acid-Schiff and Masson

Renal tissues were sectioned into 3 μm slices and stained with periodic acid-Schiff reagent (PAS) and Masson’s trichrome. The thickening of the renal basement membrane was detected by PAS staining. Six typical visual fields were selected for the statistical analysis in each mouse, and the percentage of the mesangial matrix in the glomerular area was taken as the basis for statistical scoring of glomerular injury.

Masson trichrome staining was used to assess collagen deposition in renal interstitium. The staining area (blue) in each paraffin-embedded slide was outlined using a microscope. The accumulation of blue dye area value optical density (IOD) was further analyzed by image-Pro Plus (Yibin, China) software to obtain semi-quantitative collagen deposition.

### Terminal Deoxy Nucleotidyl Transferase dUTP Nick End Labeling Assay

Left-side renal tissues were routinely prepared in paraffin-embedded sections. Paraffin sections were determined by TUNEL (terminal deoxynucleotidyl transferase dUTP nick end labeling) staining using an *In Situ* Cell Death Detection Kit (Roche), according to the instructions of the manufacturer. Five visual fields were randomly selected at high magnification to calculate the apoptosis index (AI) of the renal tubular epithelial cells [AI = (apoptotic cell number/total cell number) × 100%].

### ELISA

In renal tissue, the levels of systemic inflammation-related factors, such as NF-kB, TNF-α, IL-6, HMGB1, histone acetyltransferase (HAT), and histone deacetylase (HDAC), were measured according to the instructions of the ELISA kit.

### Real-Time Quantitative Reverse Transcription-Polymerase Chain Reaction

Total RNA was extracted according to the instructions of the TRizol KIT and was reverse transcribed into cDNA according to the instructions of the reverse transcription kit. Based on the value of the reference gene beta actin, we used the 2^–ΔΔ*Ct*^ method to analyze relative mRNA levels. The primer sequences used are listed in Table 1.

**TABLE 1:** PCR primer sequence.

GEEN	Genetic sequence	
β-actin	Forward primer	5′-CACGATGGAGGGGCCGGACTCATC-3′
	Reverse primer	5′-TAAAGACCTCTATGCCAACACAGT-3′
IL-1β	Forward primer	5′-TCAGGCAGGCAGTATCACTC-3′
	Reverse primer	5′-AGCTCATATGGGTCCGACAG-3′
IL-6	Forward primer	5′-CACAGAGGATACCACTCCCAACAGA-3′
	Reverse primer	5′-ACAATCAGAATTGCCATTGCACAAC-3′
TNF-α	Forward primer	5′-AGCACAGAAAGCATGATCCG-3′
	Reverse primer	5′-CTGATGAGAGGGAGGCCATT-3′

### Immunohistochemistry

The left kidney tissue sections were dehydrated and incubated in 3% H_2_O_2_ at 37°C for 10 min to inactivate endogenous peroxidase. Then, high-pressure antigen repair was performed. The goat serum blocking solution was sealed and incubated overnight in CD3+ (1:200), CD4+ (1:4,000), CD8+ (1:500), MPO (1:500), and HMGB1 (1:500) primary anti-antibody solutions. Paraffin sections were then incubated in the secondary antibody solution for 30 min at 37°C. Finally, DAB staining was used for color detection, and the brown particles showed positive expression under a microscope.

### Western Blot Analysis

Both whole cell protein and cytoplasmic protein were extracted from kidney tissue according to standard procedures, the protein concentration was determined using the BCA method, and the expression of renal cytoplasmic protein and whole cell protein HMGB1 was detected. After sodium dodecyl sulfate (SDS) gel electrophoresis, appropriate amounts of protein samples were mixed with a marker and transferred to the polyvinylidene fluoride (PVDF) membrane. After washing the membrane, it was sealed with skimmed milk powder and incubated overnight at 4°C with rabbit polyclonal antibody (diluted at 1:1,000). After shaking and washing again, HRP-labeled sheep anti-rabbit secondary antibody (diluted at 1:5,000) was added and incubated at 37°C for 2 h. Glyceraldehyde- 3-phosphate dehydrogenase (GAPDH) was used as the internal reference of the Western blot system. The film was exposed and scanned in the gel imaging system. The gray scale of the film was analyzed using BandScan.

### Co-immunoprecipitation

The left kidney tissue was lysed with IP lysate, and the supernatant protein was extracted, and a small amount of protein was retained for the Western blot input control. To pretreat the protein, 30 μl Agarose Protein A + G was added to eliminate non-specific binding. Then Agarose Protein A + G, co-IP antibody HMGB1 15–500 μl total protein, was removed, allowed to react with the target protein, and slowly shaken with the antigen-antibody mixture at 4°C overnight. On the second day, 30 μl Agarose Protein A + G was added for 3–6 h with a rotating apparatus at 4°C. After centrifugation, the supernatant was discarded and the precipitates were collected. Then, 30 μl 2 × SDS-PAGE electrophoresis sample loading buffer was added to resuspended precipitates. Upper clarification samples were collected for the subsequent Western blot detection. The steps of the Western blotting assay were described earlier, and the interaction between HMGB1 and acetylated HMGB1 was analyzed after obtaining the protein bands.

### Statistical Analysis

The results were expressed as mean ± standard deviation (*x* ± *s*). Independent *t*-test or one-way ANOVA was used to compare the mean of the groups. The *p*-values < 0.05 were considered statistically significant.

## Changes in the Serum Creatinine and Urea Nitrogen Levels and Analysis of Renal Pathological Damage

**FIGURE 1:**
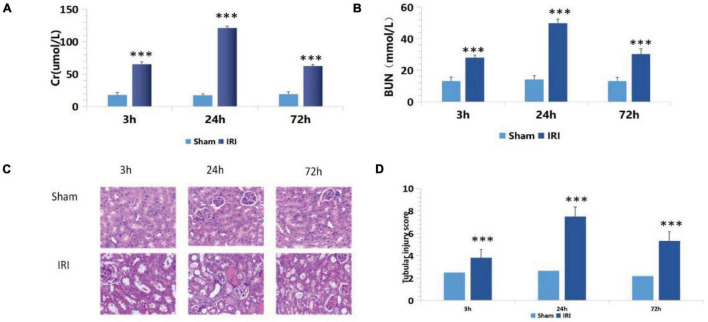
**(A,B)** De termination of serum creatinine (sCr) and blood urea nitrogen (BUN) in mice of the ischemia-reperfusion injury (IRI) group at different time points after operation; ***p < 0.001 (sham vs. IRI); **(C)** hematoxylin and eosin (H&E) staining at different time periods after renal IR in mice (200×); **(D)** Semiquantitative score of renal pathological injury in each IRI group; ***p < 0.001 (sham vs. IRI).

Compared with the sham group, the IRI group showed increased sCr levels from 17.75 ± 3.93 to 65.34 ± 3.74 μmol/L and BUN levels from 13.25 ± 2.30 to 27.87 ± 1.74 mmol/L (*p* < 0.001) 3 h after renal ischemia for 40 min. After reperfusion, they continued to increase with time, reaching peak levels at 24 h: sCr was 121.03 ± 3.03 μmol/L (*p* < 0.001) and BUN was 49.87 ± 2.61 mmol/L (*p* < 0.001). Then, they decreased gradually after 72 h of renal IR (Figures 1A,B).

There was no significant change in renal tubular epithelial cells under a light microscope in the sham group. In the IRI group, dilatation of renal tubule was observed under a light microscope for 3 h. Renal damage reached its peak at 24 h, showing glomerular swelling, extensive renal tubular lesions, disorderly arrangement, tubular epithelial cell swelling and vacuolar degeneration, epithelial cell shedding, and necrosis. Exfoliated cells accumulated in the lumen, and a large number of epithelial cell fragments and red blood cell tubules were seen in the lumen of partially expanded renal tubules. The renal interstitium was severely congested with inflammatory cell infiltrates. Renal damage reached its peak at 24 h. At 72 h after reperfusion, renal tissue damage had gradually reduced, and some renal tubular lumens were dilated, while the epithelial cells of the renal tubules were foamed off (Figure 1C).

The above semiquantitative scoring method was used to assign pathological scores to renal tissues. The results of the histopathological injury score showed that 24 h after surgery, the renal histopathological score of mice in the IRI group was significantly higher than in the sham group (Figure 1D), and the biochemical indexes of renal function showed that the damage reached the peak at 24 h after surgery. Therefore, 24 h after surgery was set as the best detection time.

## Changes in the Serum Creatinine and Urea Nitrogen Levels and Renal Pathological Damage in Mice Pretreated With Osthole

The renal function of mice improved after intraperitoneal injection of 5, 10, 20, and 40 mg/kg of osthole 30 min before renal IR. Compared with the IRI group, the renal function of the 5 mg/kg osthole preconditioning group (*p* > 0.05) was not statistically significant. As the osthole concentration increased, sCr and BUN levels gradually decreased. In the osthole pretreatment group, sCr and BUN levels in the 40 mg/kg osthole group were the lowest: sCr was 40.45 ± 4.33 μmol/L (*p* < 0.001) and BUN was 23.44 ± 1.08 mmol/L (*p* < 0.001).

**FIGURE 2:**
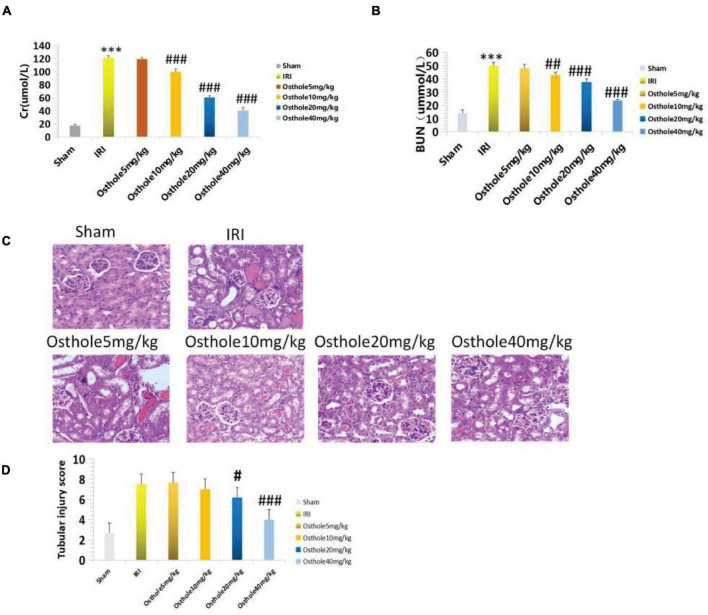
**(A,B)** Determination of sCr and BUN in mice pretreated with osthole at different doses; ***p < 0.001 (sham vs. IRI); ^##^*p* < 0.01, ^###^*p* < 0.001 (IRI vs. osthole); **(C)** H&E staining of the osthole group at different doses 24 h after surgery (200×); **(D)** Semiquantitative score of renal pathological injury in the osthole group; ^#^*p* < 0.05, ^###^*p* < 0.001 (IRI vs. osthole).

The results indicated that the difference was statistically significant. Compared with the sham group, the treatment group showed slightly increased sCr and BUN levels in the 40 mg/kg osthole group (Figures 2A,B). After intraperitoneal injection of Osthole 30 min before ischemia, renal tissue injury improved to varying degrees (Figure 2C). The results showed that the osthole treatment significantly reduced renal IR injury and further protected renal function in mice.

The pathological score of the 40 mg/kg osthole group was significantly lower than that of the IRI group (Figure 2D), and the sCr and BUN levels were the lowest. As such, the 40 mg/kg pretreatment concentration was the best.

## Periodic Acid-Schiff and Masson Staining in Each Group 24 h After Operation

**FIGURE 3:**
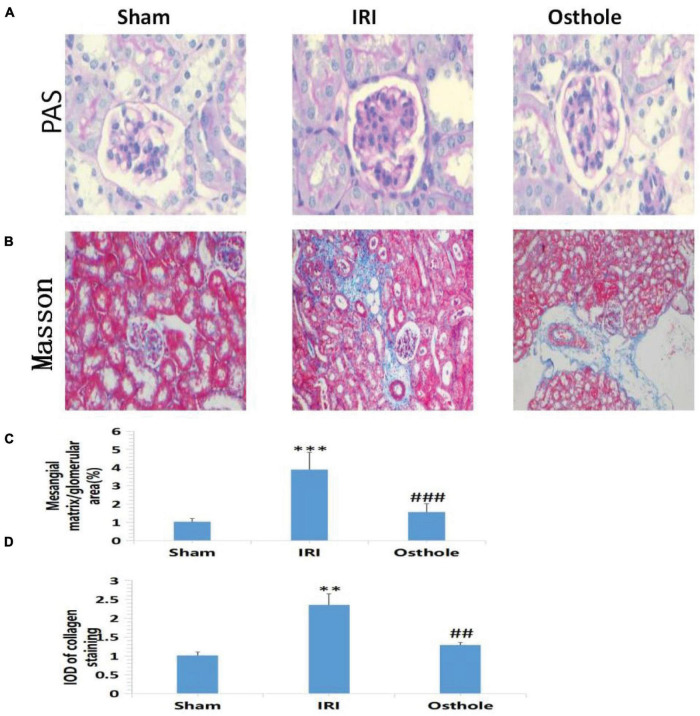
Morphological changes of renal tissues in each group 24 h after surgery. **(A,B)** PAS and Masson staining, original amplification 200×; **(C)** The percentage of the mesangial matrix in the glomerular area stained with PAS; **(D)** IOD value of collagen fiber deposition in the kidney of rats stained by Masson. The bar chart data were mean ± SD, *n* = 6. ***p < 0.001, **p < 0.01 (sham vs. IRI); ^##^*p* < 0.01, ^###^*p* < 0.001 (IRI vs. osthole).

Periodic acid-Schiff staining showed normal glomerular structure and no hyperplasia of the mesangial matrix in the sham group under a light microscope. Compared with the normal group, PAS-positive staining substances in the mesangial area of mice in the IRI group accumulated, mesangial matrix proliferated, and basement membrane thickened, and the relative area of glomerular matrix of mice in the IRI group increased significantly, with statistical significance (*p* < 0.001). Compared with the IRI group, PAS-positive stain deposition was reduced and the degree of PAS-positive stain deposition was reduced in the 40 mg/kg osthole group, and the relative area of glomerular matrix was significantly reduced, with statistical significance (*p* < 0.001) (Figures 3A,C). Masson staining results showed that collagen fibers in the sham group were neatly arranged without the obvious blue staining area. Compared with the sham group, some disordered collagen fibers and some blue staining areas around glomerulus and in renal interstitium were observed in the IRI group (*p* < 0.01), and the degree of blue staining was lighter in the 40 mg/kg osthole group, suggesting that renal fibrosis was alleviated (*p* < 0.01) (Figures 3B,D). Perhaps, due to insufficient substrate deposition time, we did not see more fiber deposition in the model group.

### Results of Terminal Deoxy Nucleotidyl Transferase dUTP Nick End Labeling Staining in Each Group 24 h After Surgery

**FIGURE 4:**
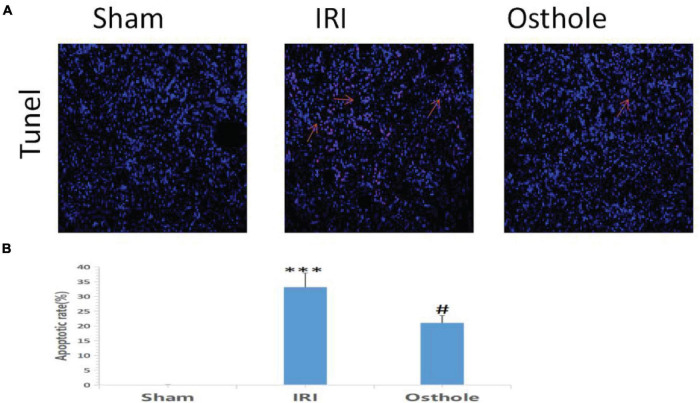
Apoptosis in the renal tissue of mice was detected by 24 h TUNEL method after the operation. **(A)** Apoptosis in renal tissue was detected using the TUNEL method with original amplification 200×. **(B)** Apoptosis rate in the renal tissue. The bar chart data were mean ± SD, *n* = 6. ***p < 0.001 (sham vs. IRI); ^#^*p* < 00.05, (IRI vs. osthole).

Compared with the sham group, TUNEL staining results showed that the IRI group exhibited an increased number of TUNEL-positive cells 24 h after surgery, and the renal tubule cells of the mice had obvious features of apoptosis (*p* < 0.001, Figure 4A). Apoptosis of renal tubule cells in mice pretreated with 40 mg/kg of osthole was improved (*p* < 0.05, Figure 4B). The differences in AI value (×10^–2^) between the IRI and sham groups and between the 40 mg/kg osthole and IRI groups were statistically significant (33.16 ± 4.85 vs. 0.057 ± 0.02, *p* < 0.001, sham vs. IRI; 20.99 ± 2.50 vs. 33.16 ± 4.85, *p* < 0.05, IRI vs. osthole).

In this study, pretreatment with 40 mg/kg osthole significantly reduced the extent of apoptosis of renal tubular epithelial cells during IRI and effectively protected renal tubular epithelial cells (*p* < 0.05).

### Expression Levels of Inflammatory Cytokines IL-6, NF-kB, TNF-α, and HMGB1 in Mice

**FIGURE 5:**
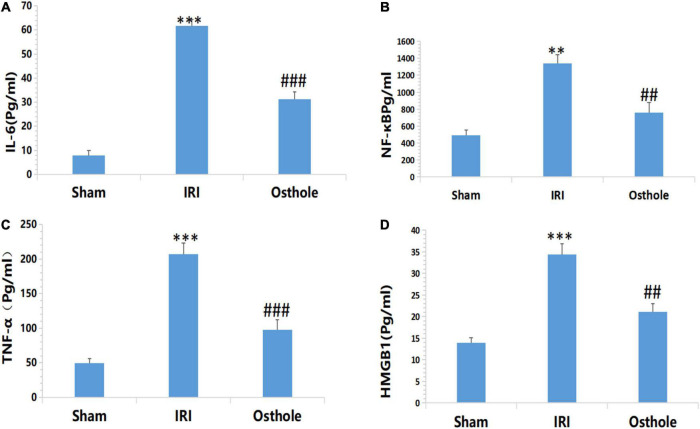
Effect of osthole on the expression of inflammatory factors in each group. **(A)** IL-6, **(B)** NF-kB, **(C)** TNF-α, **(D)** HMGB1. **p < 0.01, ***p < 0.001 (sham vs. IRI); ^##^*p* < 0.01, ^###^*p* < 0.001 (IRI vs. osthole).

Figure 5 shows that after renal IR, the expression levels of IL-6, NF-KB, TNF-α, and HMGB1 in the IRI group were significantly higher than those in the sham group. After the 40 mg/kg of osthole preconditioning, the levels of the above four indicators were all lower than those in the IRI group, suggesting that 40 mg/kg osthole can effectively reduce the production of IL-6, NF-kB, TNF-α, and HMGB1 in cases of IRI-induced inflammation.

### Expression Levels of IL-1β, IL-6, and TNF-αmRNA in Renal Tissues

**FIGURE 6:**
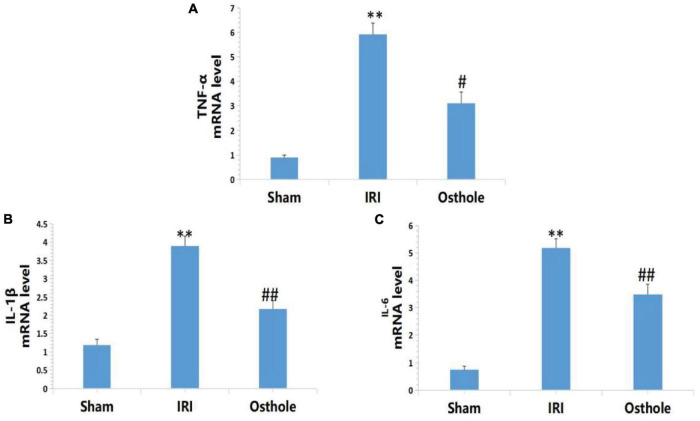
Effects of osthole on the mRNA expression of TNF-α, IL-1β, and IL-6 in each group. **(A)** TNF-α, **(B)** IL-1β, **(C)** IL-6. **p < 0.01 (sham vs. IRI); ^#^*p* < 0.05, ^##^*p* < 0.01 (IRI vs. osthole).

Figure 6 shows that the mRNA levels of TNF-α, IL-1β, and IL-6 in renal tissues in the IRI group were significantly higher than in the sham group, and the above indexes were significantly lower than those of the IRI group after early intervention using 40 mg/kg osthole. After IRI, the mRNA expression levels of inflammatory cytokines were significantly increased. Preoperative 40 mg/kg osthole pretreatment can reduce the mRNA expression levels of inflammatory cytokines, thus alleviating the inflammatory response.

### Effects of Osthole on the Expression of CD3+, CD4+, CD8 + T, and MPO Cells in Kidney Tissues

**FIGURE 7:**
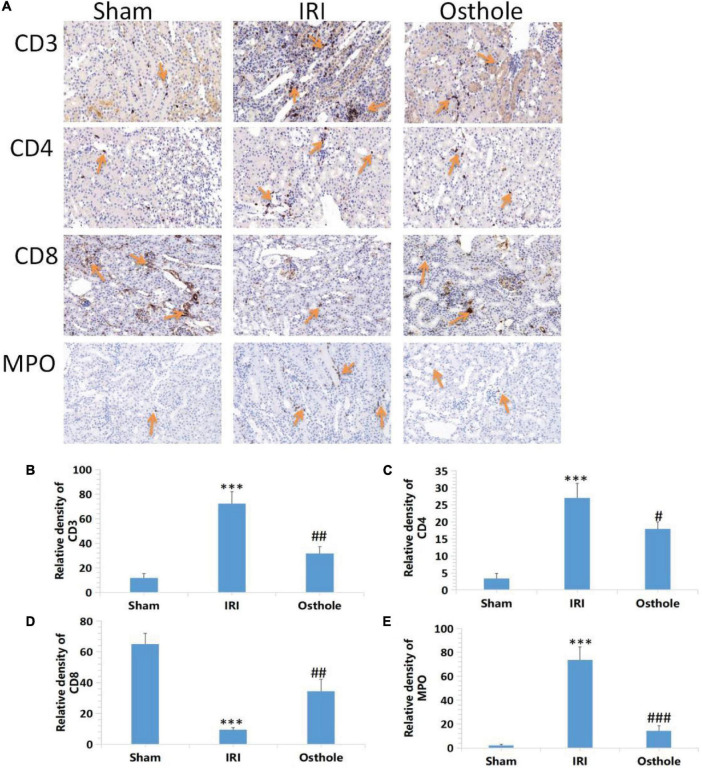
Effects of osthole on the expression of CD3+, CD4+, CD8 + T, and MPO in the kidney tissues of mice in each group. **(A)** Immunohistochemical detection of the expression of CD3+, CD4+, CD8 + T, and MPO in kidney tissues, original amplification 200×. **(B–E)** The relative density of expression levels of CD3+, CD4+, CD8 + T, and MPO in renal tissue was quantified as the change of ploidy expression relative to the control group. The bar chart data were mean ± SD, *n* = 6. ***p < 0.001 (sham vs. IRI); ^#^*p* < 0.05, ^##^*p* < 0.01, ^###^*p* < 0.001 (IRI vs. osthole).

Compared with the sham group, immunohistochemical results revealed that the IRI group showed a significantly increased proportion of CD3 +, CD4 + T, and MPO cells (*p* < 0.001), and this played a leading role in the early inflammatory response. The proportion of CD8 + T cells also decreased (*p* < 0.001). After the 40 mg/kg of osthole preconditioning, the proportion of CD3+, CD4 + T, and MPO cells was significantly decreased (*p* < 0.01, *p* < 0.05, *p* < 0.001, respectively), while the proportion of CD8 + T cells was significantly increased (*p* < 0.01), indicating that the degree of renal IRI was reduced, and the inflammatory response was weaker in the 40 mg/kg osthole preconditioning group (Figure 7).

### Effect of Osthole on the HMGB1 Expression in Renal Tissues of Mice in Each Group

**FIGURE 8:**
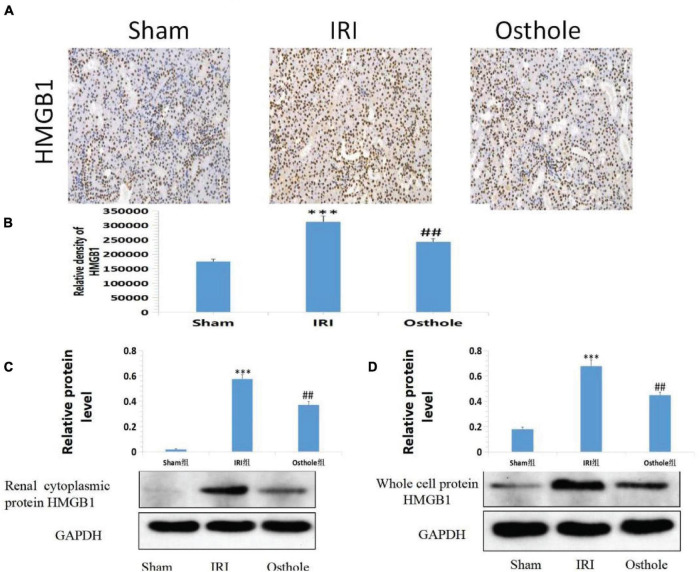
**(A)** Effect of osthole on HMGB1 expression in renal tissues of mice in each group. **(B)** The relative density of HMGB1. ***p < 0.001 (sham vs. IRI); ^##^*p* < 0.01 (IRI vs. osthole). **(C,D)** Effects of osthole on the expression of renal cytoplasmic protein and whole-cell protein HMGB1 in mice of each group and the semiquantitative statistics of the Western blot detection. ***p < 0.001 (sham vs. IRI); ^##^*p* < 0.01 (IRI vs. osthole).

In the sham group, immunohistochemical staining showed that HMGB1 was slightly visible in the renal tissue of mice that was mainly located in the nucleus. After 40 min of kidney ischemia in mice, renal tubular epithelial cell nuclei of HMGB1 were rapidly released from the nucleus into the cytoplasm (Figure 8A), and this showed that HMGB1 migrates and changes in the early stage of renal IRI and, thus, plays an early role in initiating the inflammatory cascade in the upstream of IRI inflammatory response. In mice pretreated with 40 mg/kg osthole 30 min before surgery, the migration and release of HMGB1 from renal tubular epithelial cells were significantly inhibited after renal ischemia (osthole vs. IRI group, *p* < 0.01), thereby alleviating the inflammatory response initiated by the HMGB1 migration in the early stage of ischemic injury and playing a protective role in renal IRI. Compared with the sham group, the Western blot results showed that the IRI group had a significantly increased content of the whole-cell HMGB1 (*p* < 0.001). In the 40 mg/kg osthole pretreatment group, the HMGB1 content was significantly lower than in the IRI group (*p* < 0.01), as shown in Figure 8.

Quantitative Western blot analysis of HMGB1 revealed that HMGB1 migrated rapidly from the nucleus of the renal tubular epithelial cells into the cytoplasm after IR in the kidney of mice. HMGB1 significantly increased in the cytoplasm of the renal tubular epithelial cells, and the migration and release of HMGB1 initiated the inflammatory response. In mice pretreated with 40 mg/kg osthole, HMGB1 release was significantly inhibited after ischemia injury, and the nucleoplasmic migration of HMGB1 in epithelial cells was reduced. As such, the inflammatory cascade reaction initiated by HMGB1 was weakened at the early stage of IRI, and the extent of apoptosis and necrosis of the cells of the renal tubules was reduced after reperfusion.

### Effects of Osthole on the Activities of Histone Acetyltransferase and Histone Dea Cetylase and the Acetylation Levels of Histone MGB1 and HMGB1 in Renal Tissues of Mice

**FIGURE 9:**
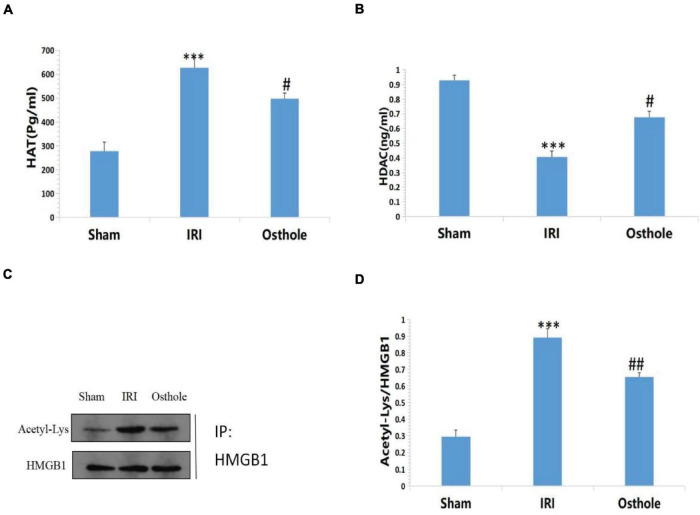
**(A,B)** Effects of osthole on HAT and HDAC activities in each group; **(C,D)** Determination of HMGB1 acetylation in renal tissue by immunoprecipitation. ***p < 0.001 (sham vs. IRI); ^#^*p* < 0.01 (IRI vs. osthole); ^##^*p* < 0.01 (IRI vs. osthole).

Compared with the sham group, Figures 9A,B shows that the IRI group exhibited highly active HAT in the renal tissues of mice (277.76 ± 37.38 vs. 626.99 ± 42.85, *p* < 0.001, sham vs. IRI), whereas in the IRI group, the HDAC in the renal tissues of mice was mildly active (0.93 ± 0.03 vs. 0.40 ± 0.04, *p* < 0.001, sham vs. IRI). Compared with the IRI group, the group with 40 mg/kg osthole preconditioning 30 min before surgery exhibited decreased HAT activity (626.99 ± 42.85 vs. 497.35 ± 23.27, *p* < 0.001, IRI vs. osthole) and significantly increased HDAC activity (0.40 ± 0.04 vs. 0.67 ± 0.04, *p* < 0.05, IRI vs. osthole). Thus, the dynamic expression imbalance of HAT and HDAC proteins after IR affected the acetylation status of histone HMGB1 in mice. These results suggest that 40 mg/kg osthole may affect the acetylation status of histone HMGB1 by interfering with the dynamic balance of HAT and HDAC activities.

Compared with the sham group, the results of immunoprecipitation reaction showed that HMGB1 had a high acetylation level in the IRI group (*p* < 0.001, Figures 9C,D), which resulted in a large amount of HMGB1 migrating away from the nucleus, causing an inflammatory reaction and leading to cell damage and apoptosis. After pretreatment with 40 mg/kg osthole, deacetylation of HMGB1 occurred in renal tissues, and the degree of acetylation of HMGB1 decreased. The results showed that 40 mg/kg osthole preconditioning interfered with the acetylation state of HMGB1 before renal IR in mice, thereby inhibiting the nucleoplasmic migration and release of HMGB1 and reducing the occurrence of renal injury. The difference between the groups was statistically significant (*p* < 0.01).

### Discussion

Renal IR is one of the major causes of renal injury. IR-associated renal injury progresses rapidly and can even lead to acute renal failure in severe cases. Currently, clinical treatment is difficult, and exploring its pathogenesis and new treatment methods is of great value.

Osthole can affect the activity of some enzymes, calcium ion transport, Na+ −K+ -ATPase activity and is widely used in the treatment of osteoporosis, lung inflammation, some skin diseases, and other diseases in traditional Chinese medicine (Basnet et al., 2001; Matsuda et al., 2002; Zhang et al., 2007; Kwak and Lim, 2014). In recent years, the protective effect of osthole on IR of tissues and organs has become a hot research topic. In addition to apoptosis, renal IR is also accompanied by inflammation, ATP reduction, and activation of various enzymes. Therefore, osthole may have a good application prospect in IR. Our previous study found that osthole can prevent renal IRI and can inhibit inflammation and reduce apoptosis to protect renal IRI by regulating PI3K/Akt signaling pathway (Xie et al., 2015).

In this study, mice were pretreated with osthole 30 min before surgery. The results showed that the sCr and BUN levels in the 40 mg/kg osthole group were significantly lower than in the IRI group. Pathophysiological changes were observed under a light microscope. In the osthole group, renal tubules were dilated; epithelial cells were shed; and necrosis, inflammatory cell infiltration, mesangial matrix proliferation, basal membrane thickening, and interstitial fibrosis were also significantly reduced in a concentration-dependent way compared with the IRI group, suggesting that osthole can reduce renal pathological changes. Furthermore, in this study, we found that osthole can reduce the expression of inflammatory cytokines NF-kB, TNF-α, IL-6, HMGB1, IL-1β, IL-6, and TNF-α in a concentration-dependent manner, change the proportion of inflammatory cells, and reduce cellular apoptosis, indicating that osthole can inhibit the inflammatory response, reduce cell apoptosis, and improve the progression and prognosis of IR, and thus protect the kidneys.

The essence of ischemic and hypoxic damaged cells will be released from the nucleus HMGBl to the cytoplasm or extracellular, activating the immune response and inflammatory response, releasing pro-inflammatory factors, and, in turn, HMGB1 itself can promote the secretion of HMGB1, thus forming a positive feedback cycle that will aggravate and amplify the inflammatory response (Wang and Ma, 2008). These inflammatory and immunological reactions induced by HMGB1 are important links in the occurrence and progression of organ IR. When released extracellular, HMGB1 acts as an early inflammatory mediator, participating in IR injury of the liver (Ilmakunnas et al., 2008), heart (Hu et al., 2016), kidney (Li et al., 2011), and other organs, as well as local and systemic inflammatory reactions (Tsung et al., 2007). Neutralizing extracellular HMGB1 with anti-HMGB1 antibodies can significantly reduce the degree of IRI. All of these studies suggest that, as an early inflammatory factor and endogenous “danger signal,” HMGB1 is involved in the inflammatory response and immune response process of renal IR, playing an important role in the commencement of reperfusion and is closely related to the occurrence or development of kidney disease (Paudel et al., 2019).

At present, targeted blocking of HMGB1 activity (e.g., an anti-HMGB1 neutralizing antibody and ethyl pyruvate) has become a new effective strategy to improve organ IRI and intervene in various acute and chronic inflammatory diseases (Yang et al., 2006; Maeda et al., 2007). However, these therapeutic strategies mainly targeted the release of extracellular HMGB1 and did not inhibit the production of HMGB1. At this point, the immune and inflammatory cascade mediated by HMGB1 may have been activated to a certain extent. Therefore, we hypothesized that whether we could block the release of HMGB1 from the nucleus to the nucleus at the early stage of IRI from the source, to play a better protective effect. Studies have shown that osthole protects against myocardial IRI in rats by decreasing the HMGB1 expression in the ischemic myocardium, and these effects may be related to its antioxidant and anti-inflammatory activities (Wang et al., 2013). Therefore, we wanted to explore whether osthole could block the release of HMGB1 from the nucleus to the nucleus from the source, thus playing a strong protective role on IRI.

During the active secretion of HMGB1, existing studies have shown that acetylation of some lysine residues on the molecule promotes the transfer of HMGB1 from the nucleus to the cytoplasm and prevents it from entering the nucleus. Acetylation of HMGB1 is the decisive regulatory mechanism for the release of HMGB1 from the nucleus to the cytoplasm (Lundback et al., 2016; Rivera Vargas and Apetoh, 2017). Protein acetylation is mainly dynamically regulated by HAT and HDAC, and a balance in the enzyme activity between HAT and HDAC may be important in the nucleation of HMGB1 (You et al., 2009; Evankovich et al., 2010). During our study, in the IRI group, the expression of HMGB1 was increased, mainly in the cytoplasm, and the expression of acetylated HMGB1 was significantly increased. The intensity of the expression and the distribution of HMGB1 were consistent with the pathological degree of renal injury, suggesting that the increased expression of HMGB1 was related to the change in the distribution, and an increase in the levels of acetylated HMGB1 was related to the occurrence and development of acute renal injury caused by ischemia and reperfusion. In the early stage of renal ischemia, a large amount of HMGB1 is released from the nucleus into the extracellular space. Osthole pretreatment can effectively block the release of HMGB1 from the nucleus into the extracellular compartment, prevent severe renal damage in mice, and significantly protect renal function. Therefore, the osthole can alleviate renal IRI by inhibiting HMGB1 migration release, and this will provide a new theoretical basis for the protective effect of osthole in renal IRI. In the IRI group, the results of this study showed that HMGB1 was in a state of hyperacetylation. HAT activity was higher while HDAC activity was lower in the IRI group than in the sham group, indicating that the high acetylated state of HGMB1 promoted the migration of HMGB1 from the nucleus to the cytoplasm, and participated in renal IRI. After osthole pretreatment, it was observed that compared with the IRI group, HAT activity decreased and HDAC activity increased. Osthole may change the acetylation state of HMGB1 and downregulate the acetylation level of HMGB1 by affecting the balance between HAT and HDAC.

In conclusion, osthole as an effective inhibitor of HGMB1 release that inhibits HMGB1 gene transcription and protein synthesis, osthole reduces the degree of acetylation of HMGB1 and the release of HMGB1 from the nucleus into the cytoplasm and extracellular space, and alleviates the cascade reaction of pro-inflammatory factors by altering the activity balance between HAT and HDAC. It can significantly reduce the pathological damage in the kidney tissues of mice and reduce renal IR damage to confer protective effects on the kidney. In this study, the pathogenesis of HMGB1 in renal IRI was explored, and a more detailed basis was provided for the preventive treatment of renal IRI using an osthole.

In future, we will explore the protective effect of osthole on the damage of primary mouse cells caused by hypoxia and oxygen stress in cell experiments, focusing on the changes of HMGB1 release, and verify the protective effect of osthole on IRI repeatedly in animal experiments. Also, we will increase the study on the pharmacokinetics of osthole *in vivo*, further enrich the research progress of its pharmacological effects, and provide a preliminary experimental basis for clinical application.

## Data Availability Statement

The original contributions presented in the study are included in the article/Supplementary Material, further inquiries can be directed to the corresponding author.

## Ethics Statement

The animal study was reviewed and approved by The Second People’s Hospital of Yibin, Yibin, Sichuan, China.

## Author Contributions

KL participated in the technical guidance of the experiment. Participated in the whole process of experimental research, data processing, and wrote the manuscript. All authors contributed to the article and approved the submitted version.

## Conflict of Interest

The authors declare that the research was conducted in the absence of any commercial or financial relationships that could be construed as a potential conflict of interest.

## Publisher’s Note

All claims expressed in this article are solely those of the authors and do not necessarily represent those of their affiliated organizations, or those of the publisher, the editors and the reviewers. Any product that may be evaluated in this article, or claim that may be made by its manufacturer, is not guaranteed or endorsed by the publisher.
