# Supplementary figures and images for: Osthole Blocks HMGB1 Release From the Nucleus and Confers Protective Effects Against Renal Ischemia-Reperfusion Injury
Source: Front Physiol. 2021 Dec 22;12:735425. doi: 10.3389/fphys.2021.735425 (PMC8727455; doi:10.3389/fphys.2021.735425)

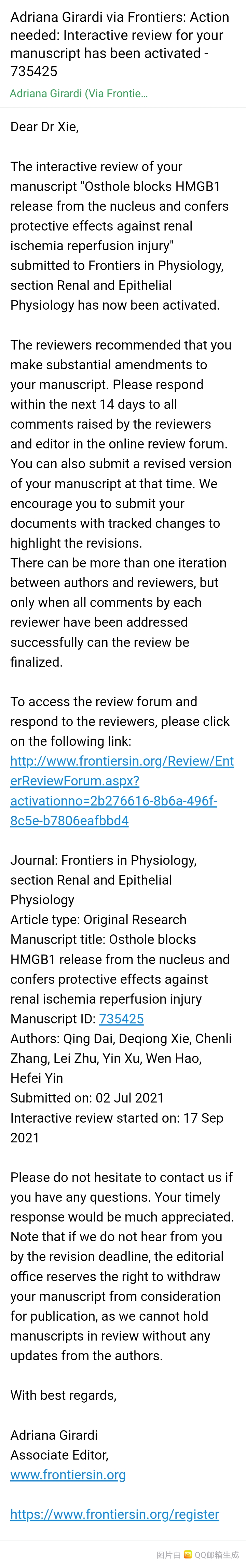

Supplement: Supplementary file 1 [file Image_1.JPEG]

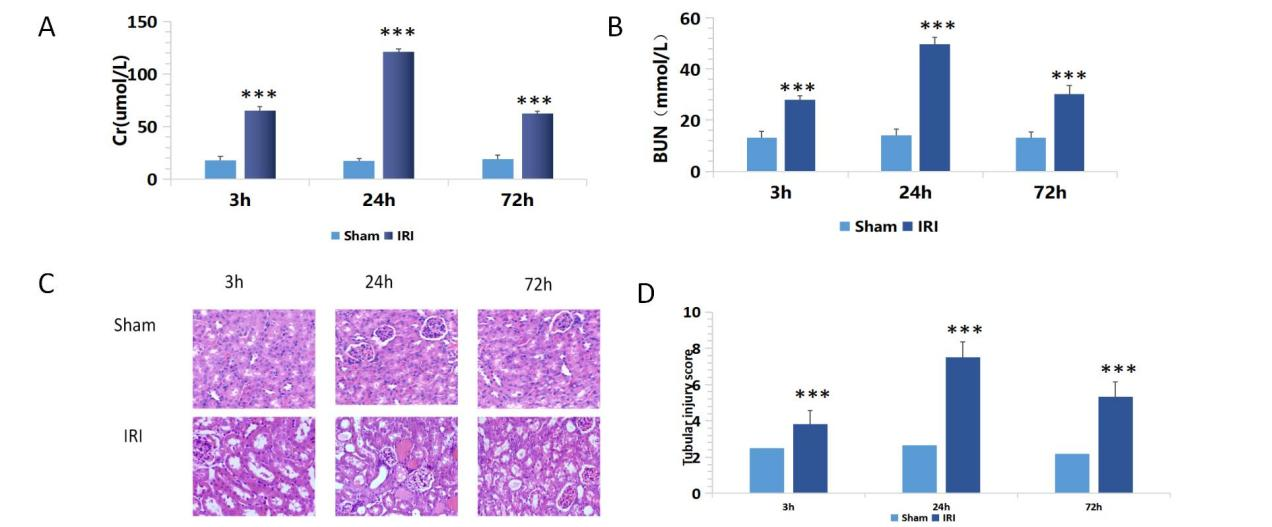

Supplement: Supplementary file 2 [file Image_2.PNG]

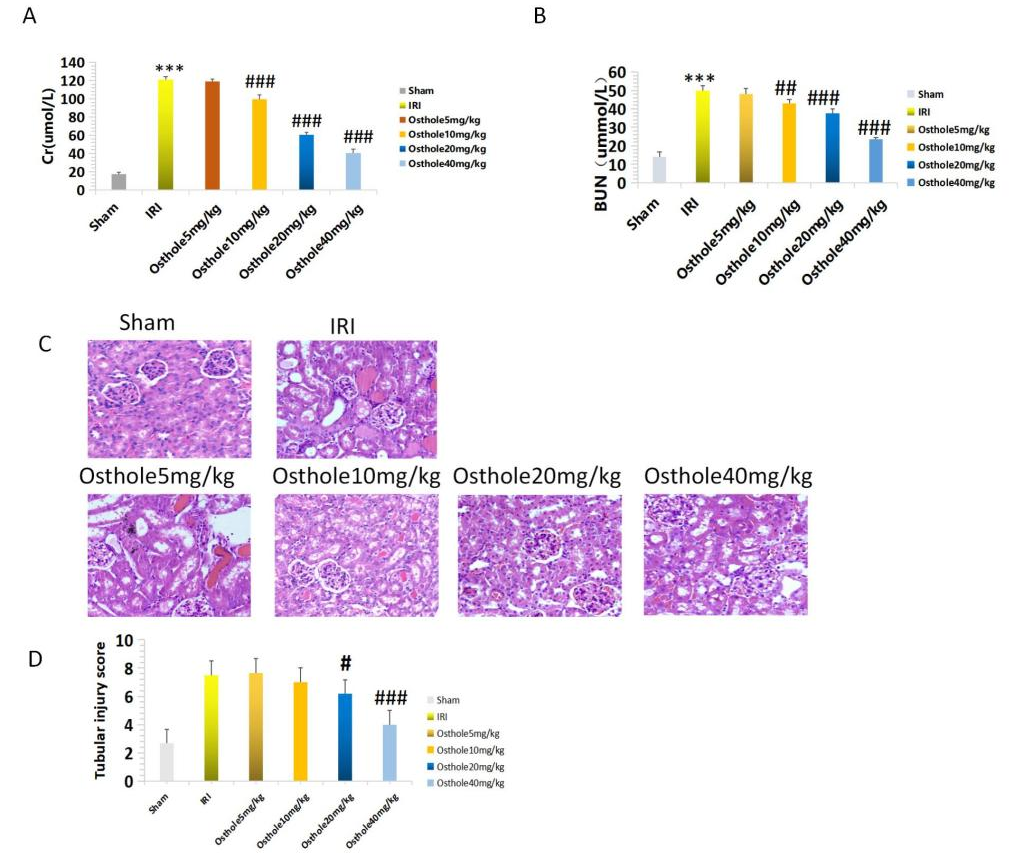

Supplement: Supplementary file 3 [file Image_3.PNG]

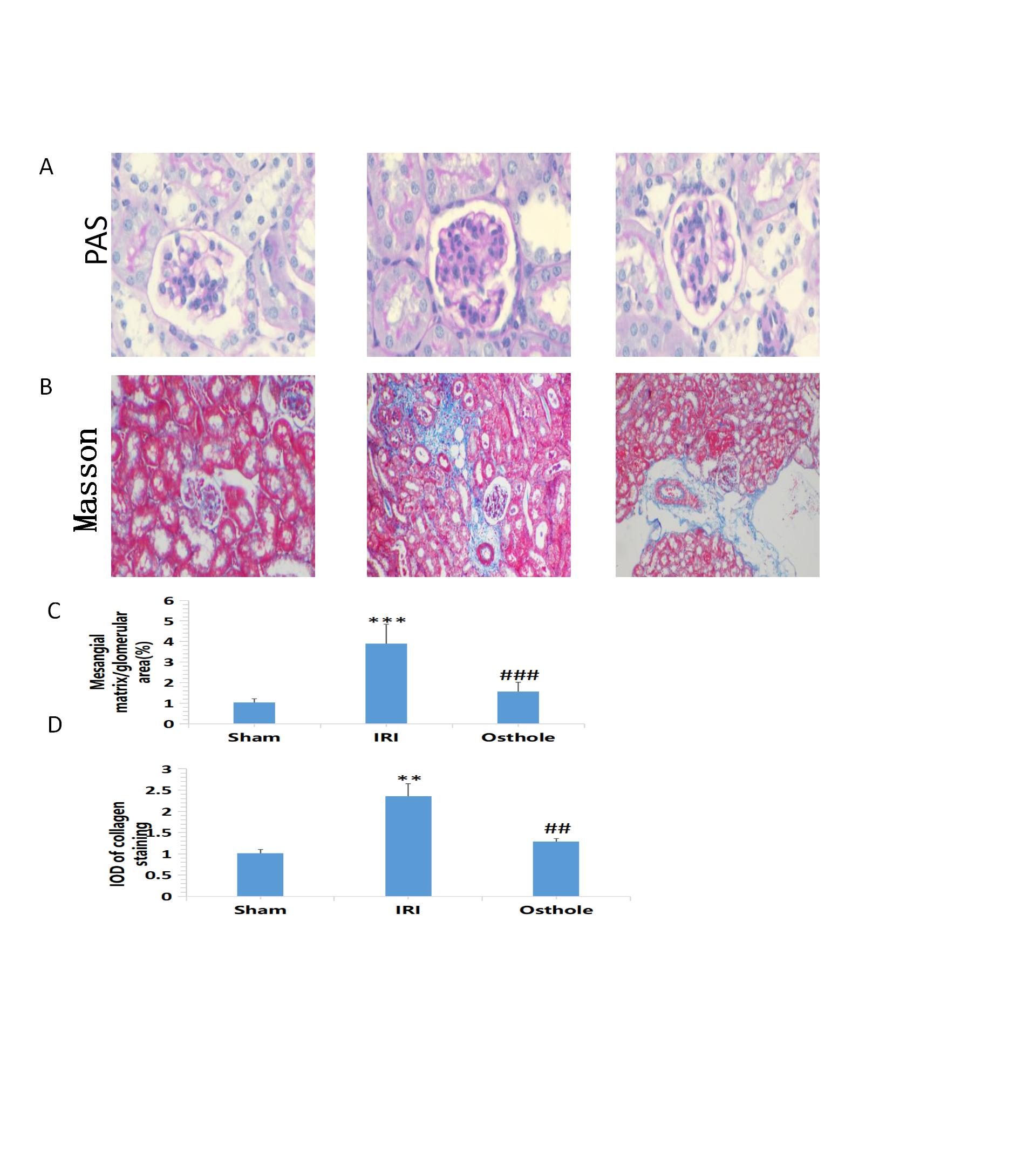

Supplement: Supplementary file 4 [file Image_4.JPEG]

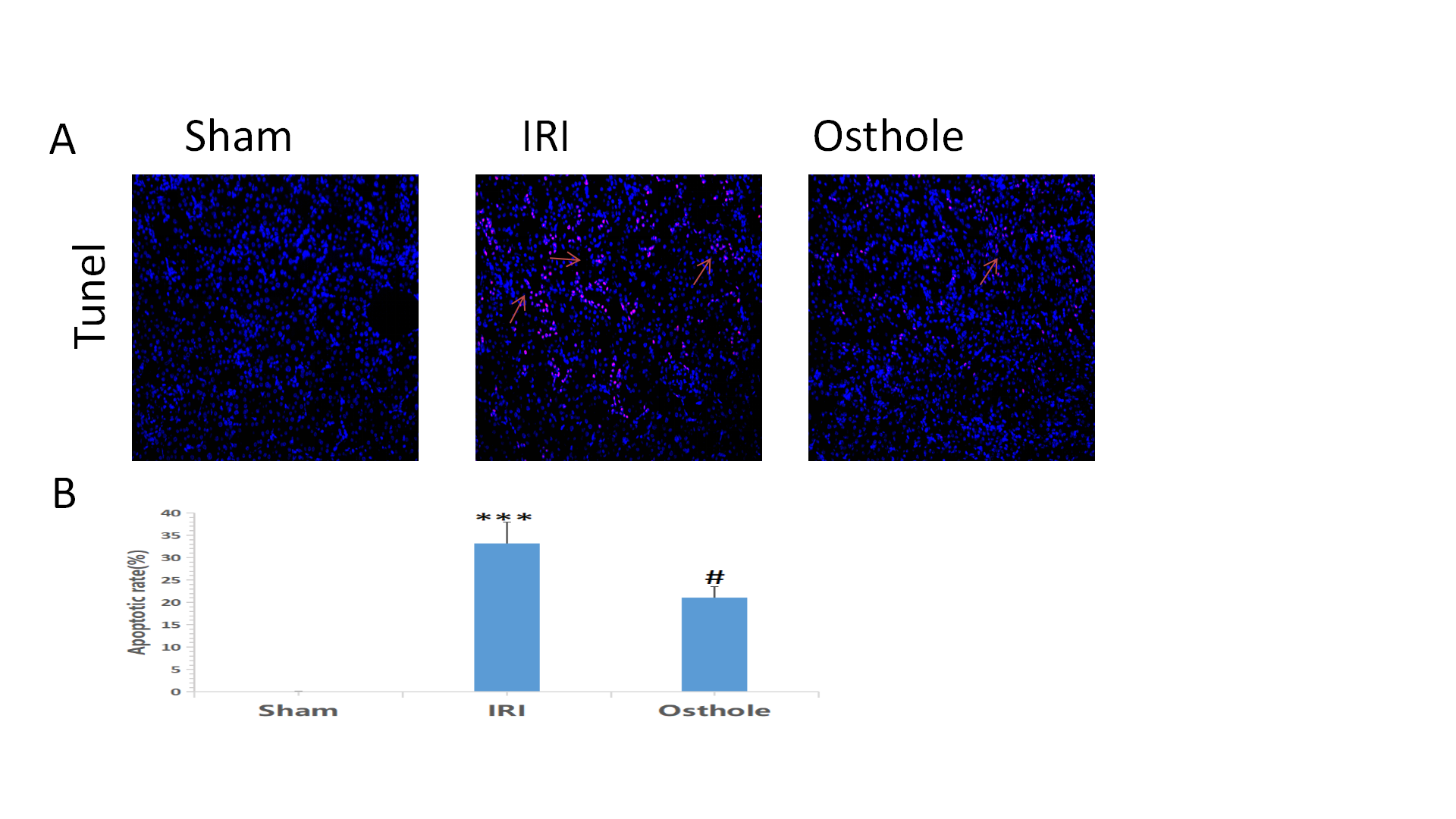

Supplement: Supplementary file 5 [file Image_5.PNG]

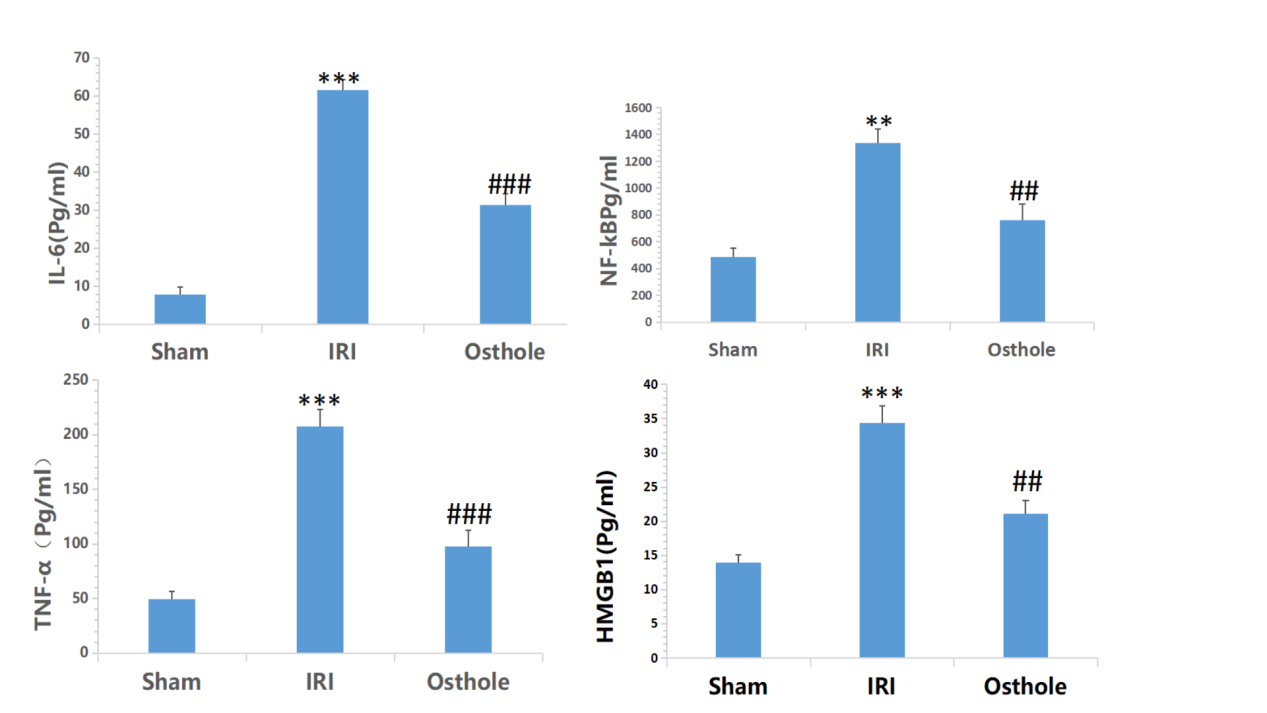

Supplement: Supplementary file 6 [file Image_6.PNG]

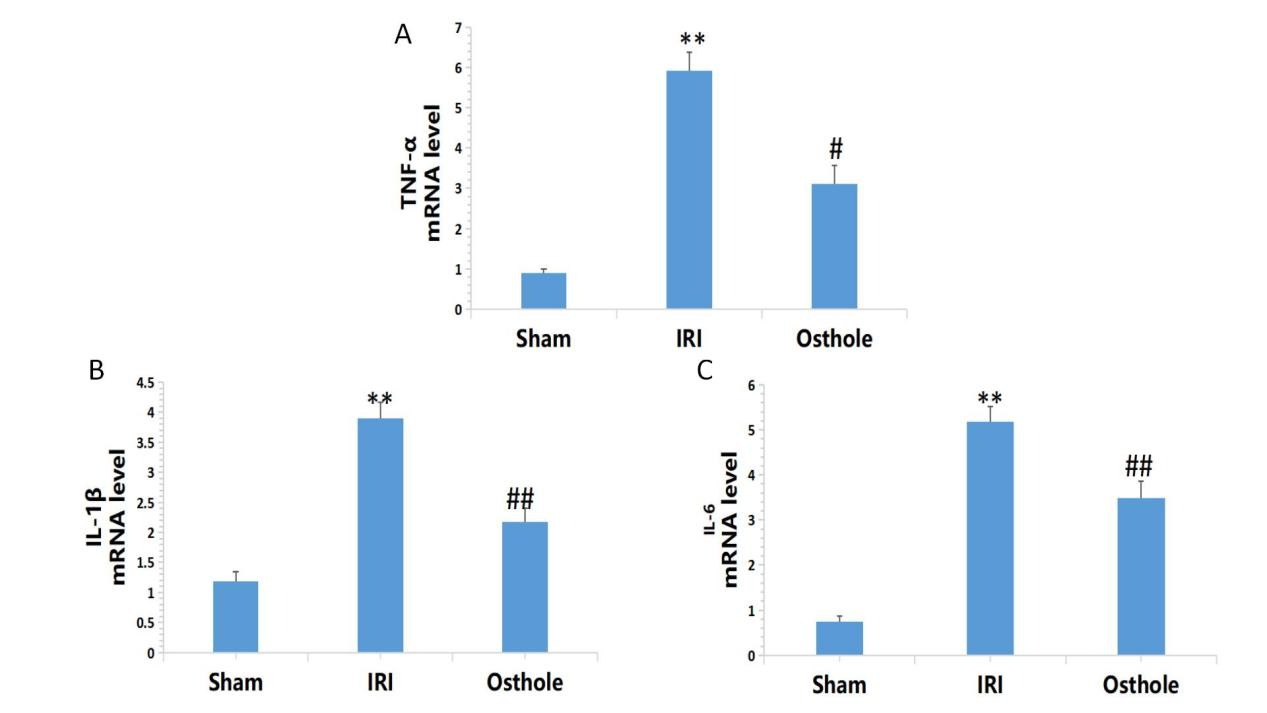

Supplement: Supplementary file 7 [file Image_7.PNG]

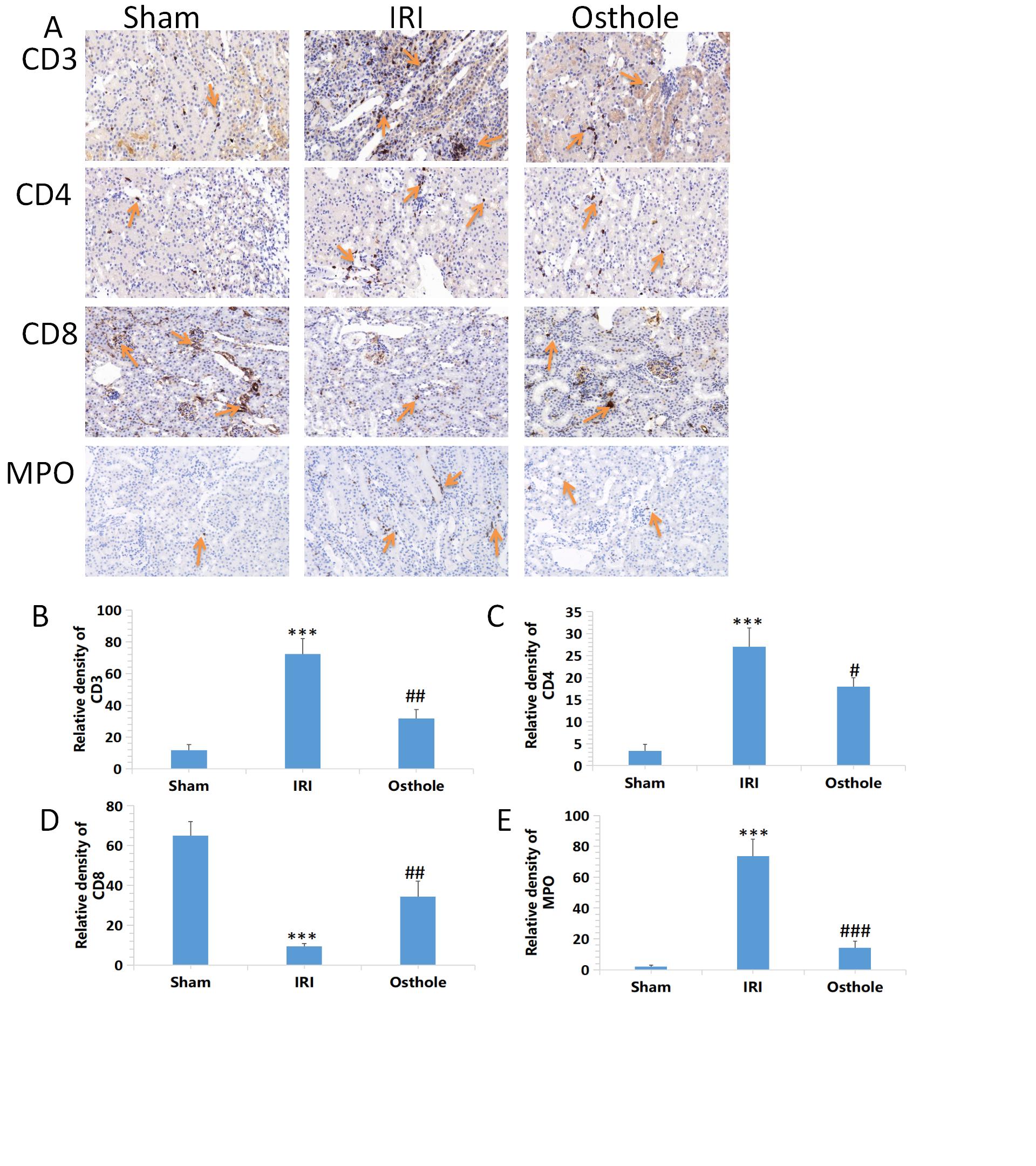

Supplement: Supplementary file 8 [file Image_8.JPEG]

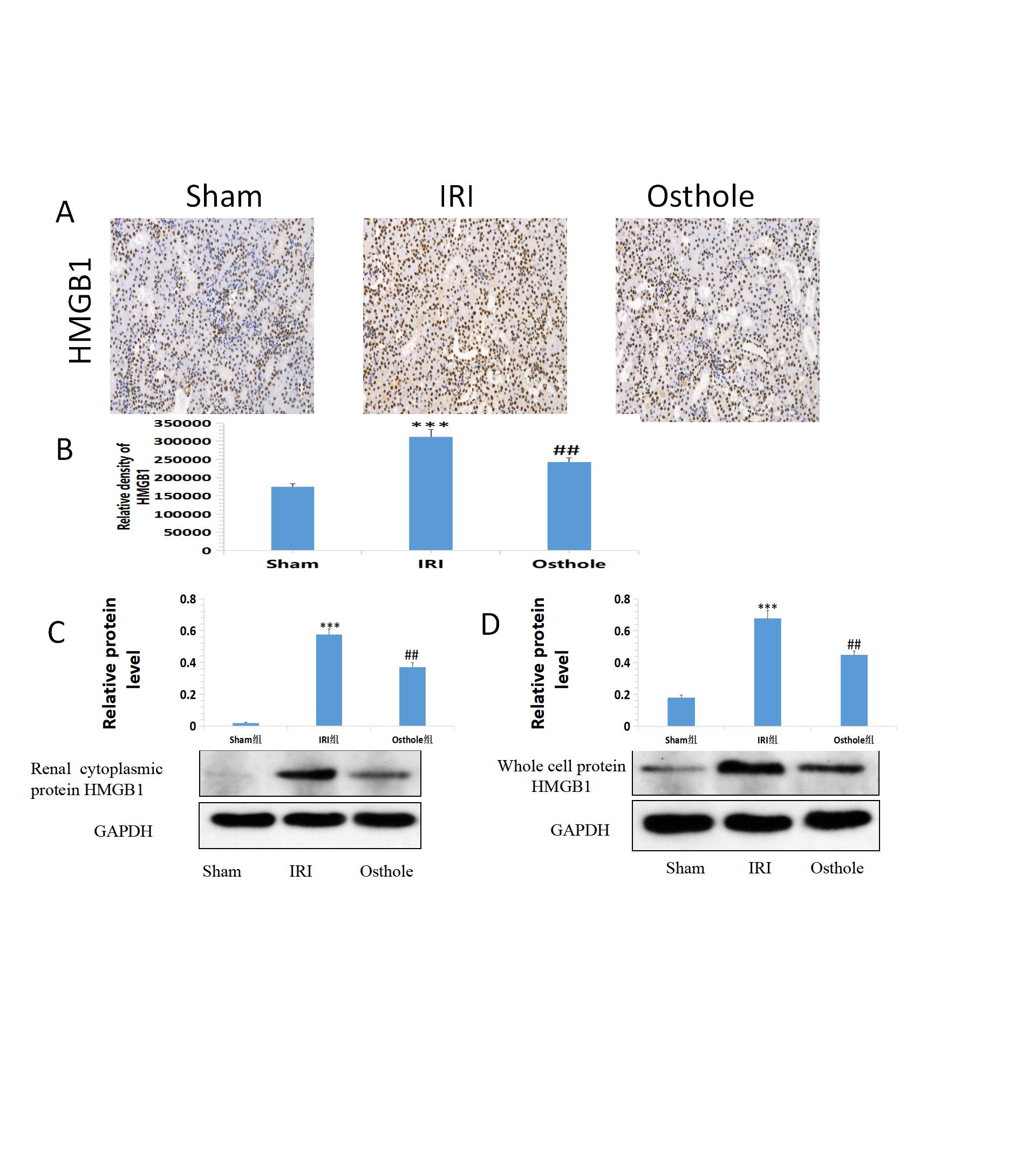

Supplement: Supplementary file 9 [file Image_9.JPEG]

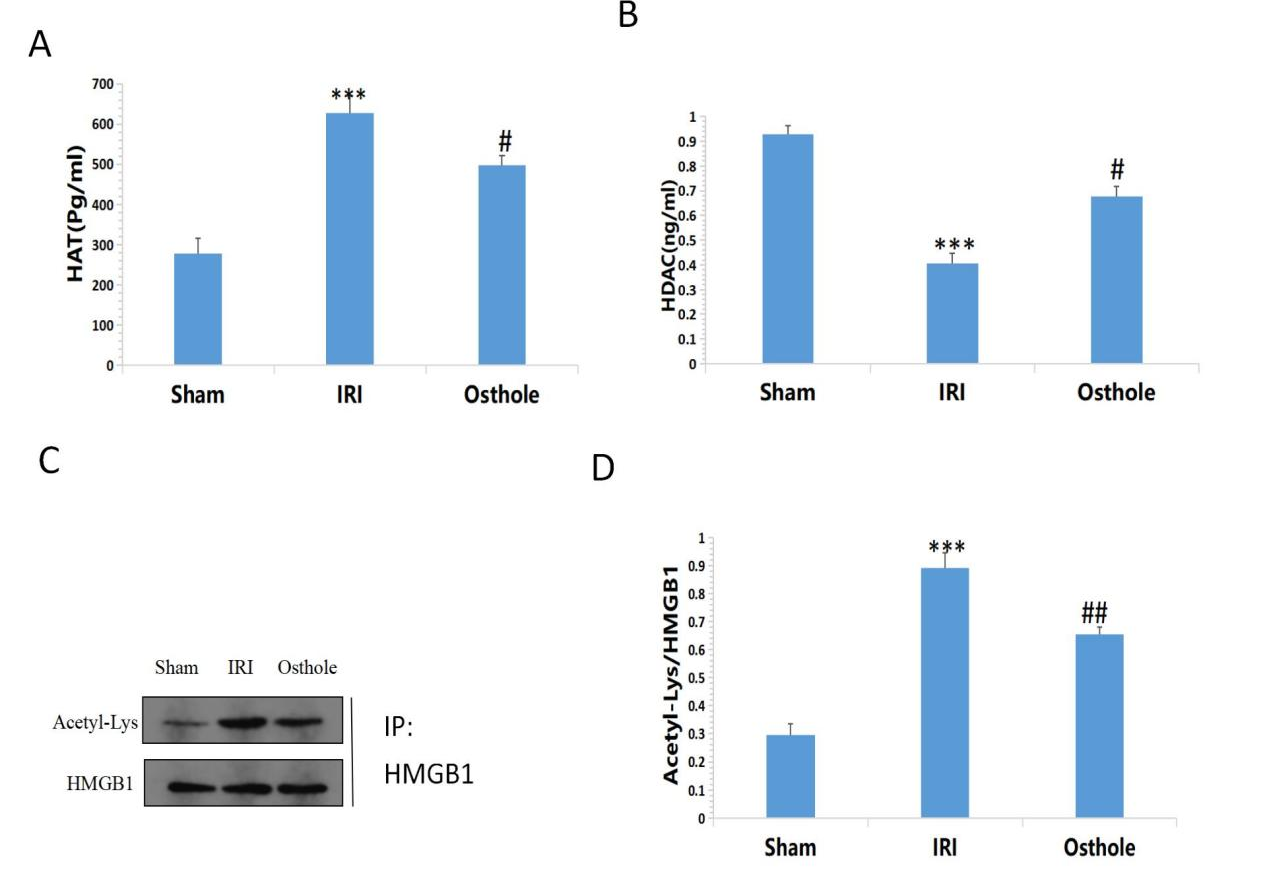

Supplement: Supplementary file 10 [file Image_10.PNG]
